# Frequency of Persistent Left Superior Vena Cava and Its Impact on Outcomes in Children Undergoing Congenital Heart Surgery

**DOI:** 10.21470/1678-9741-2024-0446

**Published:** 2025-11-07

**Authors:** Muhammet Hamza Halil Toprak, Serif Serifoglu, İbrahim Cansaran Tanıdır, Hacer Kamalı, Okan Yıldız, Eymen Recep, Sertaç Haydin, Ali Can Hatemi, Erkut Öztürk, Alper Guzeltas

**Affiliations:** 1 Department of Pediatric Cardiology, Saglik Bilimleri University Basaksehir Cam and Sakura Hospital, Istanbul, Turkiye; 2 Department of Pediatric Cardiology, Saglik Bilimleri University Mehmet Akif Ersoy Thoracic and Cardiovascular Surgery Education and Research Hospital, Istanbul, Turkiye; 3 Department of Pediatric Cardiovascular Surgery, Saglik Bilimleri University Mehmet Akif Ersoy Thoracic and Cardiovascular Surgery Education and Research Hospital, Istanbul, Turkiye; 4 Department of Pediatric Cardiovascular Surgery, Saglik Bilimleri University Basaksehir Cam and Sakura Hospital, Istanbul, Turkiye

**Keywords:** Congenital Heart Defects, Persistent Left Vena Cava Superior, Cardiac Surgery, Child.

## Abstract

**Objective:**

This study aimed to investigate the frequency of persistent left superior vena cava (PLSVC) and its impact on outcomes in children undergoing congenital heart surgery.

**Methods:**

The study was conducted retrospectively in cases diagnosed with congenital heart disease who were operated on under the age of 16 years between October 1^st^, 2021, and October 1^st^, 2024, at two major tertiary centers. The frequency of PLSVC and its possible impact on surgical outcomes were evaluated in these cases. The results were analyzed statistically.

**Results:**

There were 4,000 cases during the study period, with 52% being male. The median weight was 5.2 kg (interquartile range 4.5 - 6 kg). PLSVC was detected in a total of 260 cases (6.5%). Of these cases, 92.3% (240/260) drained into the coronary sinus, while 7.7% (20/260) drained directly into the left atrium. In 251 (96.5%) of the patients with PLSVC, there was a right SVC, while nine (3.5%) did not have a right SVC. Of the 251 patients with double SVC, 105 (42%) had a normal innominate vein. PLSVC was primarily associated with heterotaxy syndrome, atrioventricular septal defect, and vascular ring defects.

**Conclusion:**

There is an increased frequency of PLSVC among certain congenital heart disease groups, and raising awareness during echocardiographic examination can facilitate the diagnosis of PLSVC. Preoperative diagnosis of PLSVC can help in managing complications more effectively

## INTRODUCTION

**Table:** 

Abbreviations, Acronyms & Symbols				
ASD	= Atrial septal defect		LSVC	= Left superior vena cava
AVSD	= Atrioventricular septal defect		MV	= Mechanical ventilation
CHD	= Congenital heart disease		NIRS	= Near-infrared spectroscopy
CPB	= Cardiopulmonary bypass		NS	= Non-significant
CS	= Coronary sinus		PICU	= Pediatric intensive care unit
cTGA	= Corrected transposition of great artery		PLSVC	= Persistent left superior vena cava
DILV	= Double inlet left ventricle		RACHS	= Risk Adjustment for Congenital Heart Surgery
DORV	= Double outlet right ventricle		RSVC	= Right superior vena cava
ECMO	= Extracorporeal membrane oxygenation		SVC	= Superior vena cava
HLHS	= Hypoplastic left heart syndrome		TAPVC	= Total abnormal pulmonary venous connection
ICU	= Intensive care unit		TGA	= Transposition of great artery
IQR	= Interquartile range		TOF	= Tetralogy of Fallot
LCOS	= Low cardiac output syndrome		VSD	= Ventricular septal defect
LOS	= Length of stay			

Persistent left superior vena cava (PLSVC) is a common congenital anomaly of the heart. The persistence of the left anterior cardinal vein and the left horn of the sinus venosus leads to the formation of the PLSVC and the coronary sinus (CS), respectively^[1]^.

The prevalence of PLSVC is 0.3 - 0.5% in individuals in the general population and can be as high as 12% in patients with congenital heart disease (CHD)^[2]^. PLSVC is found alongside the right superior vena cava (SVC) in 80 - 90% of cases.

Mostly, PLSVC is asymptomatic and detected incidentally in diagnostic and therapeutic examinations due to different reasons^[3,4]^. However, it may be identified as a component of complex cardiac pathologies and can lead to significant clinical issues, such as potential atrial/ventricular fibrillation, due to compression of the atrioventricular node and the bundle of His caused by dilation of the CS. In unrecognized cases of PLSVC, retrograde cardioplegia - a method commonly used for myocardial protection during cardiac surgery - may be ineffective^[5]^.

Diagnostic methods to identify a PLSVC include transesophageal or transthoracic echocardiography, conventional contrast venography, computed tomography, and magnetic resonance venography^[2]^.

Literature reports on PLSVC have focused on the difficulties associated with the placement of central venous catheters or pacemakers and related complications in patients without CHD. In contrast, there are many reports associated with the rare combinations of PLSVC and CHD or with PLSVC and associated CS anomalies. The number of studies evaluating the impact of PLSVC on the perioperative and postoperative periods of cardiac surgery for CHD in children is limited^[1-3,5]^.

This study aimed to investigate the frequency of PLSVC and its impact on outcomes in children undergoing congenital heart surgery.

## METHODS

The study was conducted retrospectively in cases diagnosed with CHD who were operated on under the age of 16 years between October 1^st^, 2021, and October 1^st^, 2024, at two major tertiary centers. Prematures (< 37 weeks of gestation), patients older than 16 years at diagnosis, and patients with suboptimal echocardiographic image were excluded from the study.

The study was planned in accordance with the Declaration of Helsinki after obtaining the required approval from the local ethics committee (approval number 2024.163).

Four thousand patients were identified to have undergone surgery during this time period. Data collected included charts and echocardiography report review. All clinical, angiographic, magnetic resonance imaging, computed tomography scan, and operative data were reviewed.

Echocardiographic evaluations were performed using the Philips Affiniti 50 cardiac ultrasound system (Philips Affiniti 50 Cardiac Ultrasound, Bothell, Washington, United States of America) with a 9-MHz probe. Standard views of pediatric echocardiogram were recorded, including parasternal (long and short axis), apical (four chambers and five chambers), subcostal, and suprasternal views. Cardiac morphology was evaluated in the direction of blood flow within the framework of the segmental approach. Atrial situs, venoatrial connection (systemic and pulmonary venous return), atrium-ventricular connections, ventricles, ventricular-great artery connection, spatial position of great arteries, intracardiac defects, and extracardiac vascular anomalies were reviewed, respectively, as the main components of this approach^[6]^.

The presence of PLSVC was also confirmed by the intraoperative evaluation of the surgeons.

Outcome measures included timing of diagnosis of PLSVC (before, at, or after surgery) and timing of diagnosis of associated anomalies of the coronary sinus (before, at, or after surgery). Postsurgical outcomes were compared between children with PLSVC and a control group. Control patients were admitted to the same pediatric intensive care unit (PICU) for CHD surgery, at the same time period (range up to ± 30 days) and in the same age range (± 10%), but those without PLSVC were children without CHD. One matched control was recruited for each study patient.

Mortality, PICU length of stay (LOS), and duration of mechanical ventilation (MV) were the main outcome measures.

### Surgical Approach in the Presence of PLSVC

The type of PLSVC (isolated, presence of double SVC, drainage into the CS, or left atrium) and whether the patient has a single or biventricular physiology constitute the main basis of the surgical strategy. In patients with double SVC, the left SVC (LSVC) usually drains into the CS and often does not require intervention. However, if CS dilatation or arrhythmias develop, surgical correction may be considered. In isolated PLSVC cases where the right SVC is absent and the LSVC drains into the left atrium, systemic venous return must be safely redirected to the right atrium. This is usually achieved by anastomosing the LSVC to the right atrium or to an enlarged CS. If unilateral venous cannulation is planned during cardiopulmonary bypass, we closely monitor the adequacy of venous return and cerebral perfusion using cerebral near-infrared spectroscopy (NIRS). Especially in patients with left-sided dominant venous drainage, right SVC or right atrial cannulation may be insufficient. In such cases, we provide additional cannulation of the LSVC or apply vacuum-assisted drainage. In single ventricular surgery, the presence of a PLSVC can affect the surgical technique, timing, and hemodynamic success; therefore, venous anatomies are evaluated in detail, and surgical planning is individualized for each patient. If the innominate vein is not visible in the anatomy, we look directly to the left. If a PLSVC is present and its diameter is not very large, we close it. If cerebral NIRS does not drop, we proceed accordingly. If the innominate vein is present and upon opening the right atrium we observe significant blood flow from the CS, we check for the presence of a PLSVC. If a PLSVC is detected, we either place a cannula into it or temporarily occlude it. In single ventricular patients with an innominate vein and bilateral SVCs, we close the smaller SVC in order to perform a unilateral Glenn procedure. If both bilateral SVCs are present and large and a Glenn procedure is planned, we do not cannulate the cava. Instead, we perform the left Glenn first, followed by the right Glenn.

### Statistical Analysis

The distribution of variables was analyzed in the computer environment. Descriptive values were obtained using the IBM SPSS Statistics for Windows, version 21 (IBM Corp. Armonk, N.Y., USA) software package and expressed as median [interquartile range (IQR)] and percentage-percentile values. Pearson's chi-squared test and Mann-Whitney U test were used to compare the variables between groups. A *P*-value of < 0.05 was considered statistically significant.

## RESULTS

There were 4,000 cases during the study period, with 52% being male. The median weight was 5.2 kg (IQR 4.5 - 6 kg). Two hundred sixty children had PLSVC (6.5%), and PLSVC was identified on echocardiography before surgery in 208 patients (80%). Of these cases, 92.3% (240/260) drained into the CS, while 7.7% (20/260) drained directly into the left atrium. In 251 (96.5%) of the patients with PLSVC, there was a right SVC, while nine (3.5%) did not have a right SVC. Of the 251 patients with double SVC, 105 (42%) had a normal innominate vein. PLSVC was primarily associated with heterotaxy syndrome and atrioventricular septal defects (AVSDs). There were 18 syndromic cases: 12 with Down syndrome, five with DiGeorge syndrome, and one with Trisomy 18. Baseline data of PLSVC patients are shown in Table 1.

**Table 1:** Demographics and patient characteristics.

Variables		Median (IQR) or n%	
n		260	
Age (months)		4 (2-6)	
Weight (kg)		5.1 (3-8)	
Body surface area (m^2^)		0.30 (0.24-0.39)	
Male		140 (54)	
RACHS-1			
1-3		151 (58)	
4-6		109 (42)	
Syndrome		18 (7)	
Physiology			
Single ventricle		99 (38)	
Biventricular		161 (62)	
Situs			
Solitus		206 (79)	
Inversus		3 (1.1)	
Right atrial isomerism		27 (10.4)	
Left atrial isomerism		24 (9.5)	
Drainage site			
Coronary sinus		240 (92.3)	
Left atrium		20 (7.7)	
Presence of RSVC and bridging vein			
RSVC		251 (96.5)	
Normal innominate vein		105 (42)	
*Diagnosis*	*n*	*All patients (%)*	*PLVSC (%)*
ASD	24	24/480 (5)	9.2
Arch hypoplasia/interruption/coarctation	18	18/290 (6.2)	6.9
AVSD	27	27/208 (13)	10.4
TGA	8	8/200 (4)	3
Pulmonary atresia	20	20/370 (5.4)	7.7
DORV	18	18/195 (9.2)	6.9
HLHS	12	12/120 (10)	6.9
Vascular ring	4	4/16 (25)	1.6
Pulmonary valve stenosis	2	2/20 (10)	0.8
Tricuspid atresia	6	6/80 (7.5)	2.4
TAPVC	4	4/70 (5.7)	1.6
Tetralogy of Fallot	15	15/309 (4.9)	5.8
Truncus arteriosus	2	2/20 (10)	0.8
DILV	5	5/41 (12.1)	1.9
VSD	37	37/1295 (2.8)	11.9
cTGA	2	2/40 (5)	0.7
Heterotaxy syndrome	51	51/116 (43.9)	19.6
Other	5	5/140 (3.6)	1.9
Total	260	260/4000 (6.5)	100

ASD=atrial septal defect; AVSD=atrioventricular septal defect; cTGA=corrected transposition of great artery; DILV=double inlet left ventricle; DORV=double outlet right ventricle; HLHS=hypoplastic left heart syndrome; IQR=interquartile range; PLVSC=persistent left superior vena cava; RACHS=Risk Adjustment for Congenital Heart Surgery; RSVC=right superior vena cava; TAPVC=total abnormal pulmonary venous connection; TGA=transposition of great artery; VSD=ventricular septal defect

The clinical characteristics of the PLSVC and control groups are shown in Table 2. Compared with the control group, the PLSVC group was significantly younger. There were no statistical differences in bypass time, Risk Adjustment for Congenital Heart Surgery score repartition, duration of MV time, and PICU LOS between both groups. The percentage of extracorporeal membrane oxygenation (ECMO) use (8.1% *vs.* 3%; *P* = 0.03) and the development of chylothorax (7.7% *vs.* 1.3%; *P* < 0.001) were higher in the PLSVC group. Although mortality was not statistically significant, it was higher in the PLSVC group compared to the control group (8.4% *vs.* 6.9%).

**Table 2:** Clinical variables for patients with PLSVC and control patients.

Variables	PLSVC (+) (n = 260)	Control (n = 260)	*P*-value
Age, months	3 (2 - 4)	6 (3 - 9)	0.02
Weight, kg	5.1 (3 - 8)	7.2 (6 - 8.4)	0.040
Male	140 (54)	132 (51)	NS
Single ventricle physiology	99 (38)	94 (36)	NS
Cyanotic heart disease	143 (55)	130 (50)	NS
Duration of preoperative mechanical ventilation, days	2 (0 - 4)	1 (0 - 2)	NS
CPB use	33 (91.6)	68 (94.5)	NS
CPB time, min	80 (65 - 95)	70 (60 - 80)	NS
RACHS-1 ≥ 4	109 (42)	104 (40)	NS
Duration of postoperative mechanical ventilation, days	3 (1 - 5)	3 (1 - 5)	NS
ECMO	21 (8.1)	8 (3)	0.03
Arrhythmias	22 (8.3)	25 (9.7)	NS
Acute kidney injury	43 (16.6)	28 (11.1)	NS
LCOS	78 (30.5)	65 (25)	NS
Chylothorax	20 (7.7)	3 (1.3)	< 0.001
ICU stay (days)	6 (4 - 8)	5 (3 - 7)	NS
Postoperative hospital stay (days)	13 (10 - 16)	12 (8 - 14)	NS
Mortality	22 (8.4)	18 (6.9)	NS
Median (interquartile range), n (%)			

CPB=cardiopulmonary bypass; ECMO=extracorporeal membrane oxygenation; ICU=intensive care unit; LCOS=low cardiac output syndrome; NS=non-significant; PLSVC=persistent left superior vena cava; RACHS=Risk Adjustment for Congenital Heart Surgery

## DISCUSSION

This study investigated the frequency of PLSVC and its impact on outcomes in children diagnosed with CHD who underwent surgery at two high-volume cardiac centers. PLSVC is not an uncommon finding in children with CHD; it can be highly associated with heterotaxy syndrome and AVSD and may include various anatomical variants without affecting postoperative outcomes. It is important to identify these associated lesions preoperatively in cases of complex or simple congenital malformations such as single ventricle, septal shunts, or extracardiac shunts. Given the results of our study and the number of cases evaluated, it represents one of the largest series in the literature on this topic.

In the literature, the prevalence estimates of PLSVC in children with CHD have been reported to range widely from 1.3% to 11%^[2,7]^. These wide ranges reflect the small cohorts and selection bias in published series, as participants typically include only those who have undergone catheterization or surgery, pathology studies, or fetopsy^[2,3]^. Considering two recent large series studies, Perles et al.^[1]^ reported a prevalence of 2.9% for PLSVC in 8,140 cases of CHD evaluated by echocardiography, while Ari et al.^[5]^ reported a prevalence of 3.3% in 2,663 cases evaluated only by cardiac catheterization and angiography^[1]^. In our study, which included only surgically treated cases, the prevalence of PLSVC was 6.5%.

In 90% of patients with PLVSC, drainage occurs into the right atrium via the CS. In contrast, in 10% of patients, it typically drains into the left atrium through an unroofed CS, or in rare cases, directly or via the pulmonary vein into the left atrium^[2]^. In the study by Ari et al.^[7]^, all drainage was observed to occur into the right atrium via the CS. In the series by Poncini et al.^[8]^, PLSVC was associated with a partially or completely unroofed CS (17%) and with CS ostial atresia (4%). In our study, the rate of drainage into the CS was 92.3%.

Echocardiography is a practical and non-invasive method for evaluating the structure of the heart. CS is widely observed in the echocardiography of patients in whom PLSVC drains to the CS. However, it has been stated that in some cases, PLSVC may not be visible with echocardiography^[3]^. In the series by Poncini et al.^[8]^, they were able to detect 83% of cases with PLSVC through preoperative echocardiography in patients diagnosed with CHD who underwent surgery. In our study, this rate was found to be 80%. Our data indicated that PLSVC can be overlooked with echocardiography, and therefore, in all cases, the pediatric cardiologist should be aware of different signs that could raise suspicion of PLSVC.

To date, many cardiac anomalies associated with PLSVC have been described and classified in various ways. The main groups of cardiac anomalies include shunt lesions, conotruncal malformations, left-sided obstructive lesions, right-sided lesions, and single ventricle anomalies^[1-3]^. Additionally, there is a wide range of information in the literature regarding the frequency of cardiac anomalies associated with PLSVC. Cha et al.^[9]^ reported that the most frequently associated anomaly is ASD. According to Eldin et al.^[10]^, complete AVSD ranks first. In the studies of Nagasawa et al.^[3]^, the high incidence group included coarctation of the aorta (23.7%) and double outlet right ventricle patients (24.6%). According to Lendzidan et al.^[11]^, the most common cardiac anomalies accompanying PLSVC are single ventricle, AVSD, and tetralogy of Fallot (TOF). In our study, the most commonly observed associated cardiac anomalies were heterotaxy syndromes and AVSD.

The clinical significance of a PLSVC depends on its drainage site and associated anomalies. PLSVC without accompanying cardiac anomalies is usually asymptomatic and is often identified incidentally. In cases where PLSVC drains into the right atrium, the CS is typically dilated. This dilation may compress the atrioventricular node and the bundle of His, potentially leading to cardiac arrhythmias. Compression of the left atrium and reduced cardiac output may also occur due to this enlargement. Furthermore, the presence of CS dilation can complicate mitral valve surgery due to its close anatomical relationship. Awareness of PLSVC prior to central venous catheter placement can be helpful^[1-6]^. The presence of CS ostial atresia is also critical in operations requiring PLSVC ligation. In this case, the CS still drains blood from the coronary veins to the right atrium via the retrograde PLSVC-left brachiocephalic vein-right SVC pathway instead of the atretic ostium. Ligation of the PLSVC in this situation would be catastrophic due to the acute interruption of cardiac venous drainage. Left atrial drainage of the PLSVC may sometimes remain asymptomatic, as it does not cause a significant right-to-left shunt. When the shunt leads to more prominent desaturation, it manifests as severe cyanosis, syncope, reduced exercise tolerance, and progressive fatigue. Thromboembolic events and even brain abscesses may occur in these patients. Treatment in such cases depends on the anatomy: if a bridging vein of adequate size is present, the PLSVC can be ligated; if the bridging vein is inadequate or the right SVC is absent, reanastomosis of the PLSVC to the CS may be required. Knowledge of PLSVC presence is fundamental in venous redirection procedures, surgeries involving cavopulmonary anastomoses (Glenn, Fontan), and heart transplantation. In unrecognized cases of PLSVC, retrograde cardioplegia - a commonly used method for myocardial protection during cardiac surgery - will be ineffective. To prevent retrograde flow, clamping of the PLSVC may be necessary. However, due to the steal effect of the hemiazygos venous system connected to the PLSVC, cardioplegia may still fail even after clamping. During cardiopulmonary bypass, unawareness of PLSVC may result in excessive venous return from the right atrium and insufficient venous return to the pump. This issue is commonly observed in pathologies such as pulmonary atresia, tricuspid atresia, and TOF. In such cases, increased systemic venous pressure can exceed left atrial pressure. In the literature, reports or series of cases describe the potential problems due to PLSVC and associated anomalies before, during, and after congenital cardiac surgery^[1,2,12,13]^. Filippini et al.^[14]^ reported the problematic association of Glenn and Fontan palliation with associated PLSVC. Santoscoy et al.^[15]^ reported that the coexistence of CS atresia and PLSVC may potentially lead to myocardial ischemia and necrosis during the repair of other associated cardiac anomalies. Poncini et al.^[8]^ evaluated the impact of PLSVC on surgical outcomes in 371 cases. The results of the study show that PLSVC in association with congenital cardiac malformation increases the risk of mortality in children undergoing cardiac surgery with cardiopulmonary bypass. In our study, we found that PLSVC did not increase mortality, but the use of ECMO and the development of chylothorax were significantly higher in cases with PLSVC. The more recent nature of our study compared to others, along with technical advancements and increased awareness of PLSVC, may explain why mortality and morbidity were not affected.

### Limitations

The main limitation of this study is that it was conducted retrospectively. The fact that surgeries were performed by different surgical teams may have influenced the postoperative outcomes. The increased frequency of ECMO use and the development of chylothorax in patients with a PLSVC may reflect the influence of associated congenital cardiac anomalies particularly heterotaxy syndrome as well as the younger age commonly observed in the PLSVC group.

## CONCLUSION

PLSVC is not an uncommon finding in children with CHD. Most often it does not affect cardiac surgery or postoperative outcomes. There is an increased frequency of PLSVC among certain CHD groups, and raising awareness during echocardiographic examination can facilitate the diagnosis of PLSVC. Preoperative diagnosis of PLSVC can help in managing complications more effectively.

## Data Availability

The authors declare that the data will only be available upon request to the authors.

## Artificial Intelligence Usage

The authors declare that no artificial intelligence tool was used in the preparation of this article.
